# School Smoking Policies and Health Science Students' Use of Cigarettes, Shisha, and Dipping Tombak in Sudan

**DOI:** 10.3389/fpubh.2019.00290

**Published:** 2019-10-11

**Authors:** Salma El Tayeb El Amin

**Affiliations:** Faculty of Social Sciences, Health Sciences, Tampere University, Tampere, Finland

**Keywords:** school smoking policy, residential smoking, tobacco, smoking, tombak, shisha, water-pipe

## Abstract

The relationship between school smoking policies and students' tobacco use is ambiguous, and little is known about the effect of these policies in low- and middle-income countries. This study was designed to assess the effects of schools' smoking policies and the exposure to residential smoking on cigarette smoking and the use of different kinds of tobacco products by Health Science students. Self-reports of cigarette smoking, use of shisha (smoking of fruits-mixed tobacco using a bowl and a connected hose); dipping tombak (local smokeless tobacco that users usually place inside oral cavity in the groove behind the lower lip), and tobacco use on school premises are analyzed. A cross-sectional survey was carried out using a modified self-report questionnaire, originally developed by WHO, among a representative sample of 1,590 third-year HSS from 25 schools drawn from 13 universities, using a multi-stages sampling technique. The response rate was 100% for schools and 68% for students. A multilevel analysis was performed by nesting student-level in school-level variables. Results from the adjusted models revealed that, when students reported awareness of smoking restriction, they were more likely to be current smokers (OR = 2.91; 95% CI: 1.68–5.02; *p* = 0.021) and shisha users (OR = 2.17; 95% CI: 1.54–3.06; *p* = 0.021). Results from additional analysis performed among tobacco users only, showed increased risk of smokers and tombak dippers who smoked or dipped on school premises (OR = 2.38; 95% CI: 1.34–4.25; *p* = 0.003, OR = 2.60; 95% CI: 1.22–5.56; *p* = 0.013, respectively). Current smokers (OR = 3.12; 95% CI: 1.98–4.92; *p* = ≤ 0.001), ever smokers (OR = 1.66; 95% CI: 1.31–2.10; *p* = ≤ 0.001) and shisha users (OR = 1.73; 95% CI: 1.36–2.21; *p* = ≤ 0.001) were exposed to residential smoking on one or more days during the previous 7 days. High percentages of those who used any kind of tobacco products reported being aware of school smoking policies, indicating no clear evidence that school smoking policies had an effect on use of any of the mentioned tobacco products. The lack of compliance with school policies shows the need for further policy enforcement and sustainability, taking into account the effect of residential smoking and social influences.

## Introduction

Tobacco use is one of the greatest challenges to public health, with steadily increasing consumption and a rapidly growing epidemic in the low- and middle-income countries (1). It has been well documented with overwhelming scientific evidence that trends of tobacco product use are increasing for some sub-groups within the global population, and its effect on health is undeniable. The available data demonstrate that about one quarter of the world's adult population currently smokes, and several million people become fatally addicted every year (2–4). Most documented successes in reducing tobacco use have occurred in developed countries by effective tobacco control policies (5, 6). In contrast, most of the developing countries require more efforts to reach a comparable level. Despite the evidence, some policy-makers in low-income countries have failed to regard tobacco use as a health priority and do not fully appreciate the essential influence of public policies related to tobacco control (7). This lack of understanding is one partial explanation for the near absence of adequate prevention measures in these countries (8).

The problem is particularly challenging in Africa, which presents a great risk in tobacco use. Cigarettes are becoming increasingly affordable, and there are limited strategies to combat the increasing trends in use of shisha (1, 9), use of smokeless tobacco (10), and their harmful effects on future morbidity and mortality (1, 11). This shift to developing settings is partly because of the global tobacco industry fierce marketing strategies and partly due to lack of adequate tobacco control measures (7, 12, 13). In Sudan, as one example of a sub-Saharan African country, smoking cigarettes, shisha use, and tombak dipping are widely spread among adolescents and young adults (14, 15).

Schools are particularly important and considered to be the best places where students' tobacco use can be targeted. Schools represent a key environment where prevention and control strategies can be implemented (16, 17). Anti-smoking policies can reduce the prevalence of smoking by promoting prevention, restriction, cessation, and by preventing students from using tobacco on school premises and protecting non-smokers (18–20). Well-known statements document that smoking behavior is mainly recognized and established at or before the age of 18 (21). Consequently, smoking prevention programs have been focused on adolescents, mainly within school settings. Other researchers have suggested that college students might be an important audience in initiation and in the development of regular smoking (22), whatever the age of the students. In addition to schools, a better understanding of the main factors that affect students' tobacco habits in other environments would be useful for policy formulation in tobacco control programs. For instance, exposure to smoking in students' neighborhoods and residential areas can have an important role in their tobacco use (23).

Regardless of the methodological issues or the heterogeneity of exposure definitions in observational studies, various researchers have evaluated the effectiveness of school tobacco policies (20, 24). Some of them revealed a significant association between student smoking on school premises and weak policy (25). Others concluded that perceptions of students' smoking on the school premises also varied according to enforcement of smoking restrictions (20, 26).

These policies are particularly important in Health Science students' schools, where students are trained and seeking careers in the health professions. Ideally, they will be role models for patients. The extent to which they effectively guide tobacco users may largely depend on their own tobacco use behavior (27). In Africa, doctors are regarded as the most likely persons from whom advice about tobacco use would be accepted by both users and non-users. Very brief advice from the doctor yields positive 1-year quit rates (28).

Within a multilevel context, the study reported here was designed to examine the effects of university policies and residential smoking on smoking and the use of different kinds of tobacco products by students studying in the Health Science disciplines of medicine, dentistry, nursing, and pharmacy. Third-year students were asked to self-report their use of cigarettes, tombak dipping, and shisha, as well as their awareness of school policies that banned or restricted use of tobacco products, and their exposure to smoking in places where they live.

## Methods

The current study used cross sectional collection of data as part of the Global Tobacco Surveillance System (GTSS) 2007. Full details of the surveys can be found elsewhere (*Centers for Disease Control and Prevention (CDC) 2006*) (29). Newer data, targeting the same student population, can not be obtained because the conditions of the country have changed, Sudan split into two countries.

A multi-stage cluster sampling design was used in the first part of the sampling technique*. The first stage*: from a total number of 39 public and private universities, 13 universities were selected based upon having a school of medicine, dentistry, nursing, or pharmacy, or any combination of these four schools (one university might have one or more Health Science schools). The rest, 26 universities, were eliminated because they did not meet the selection criteria. *The* second *stage*: all Health Science schools (*N* = 29) were drawn from the 13 universities. Four of the 29 Health Science schools were eliminated because they were new and did not have a third class at the time of the survey. Thus, the final sample became 25 schools (Medicine 10; Dentistry 6; Nursing 4; and Pharmacy 5). The third year classes in the 25 schools were selected purposively. All students who were enrolled and present in these classes, regardless of their age, were eligible to participate in the study.

The Health Science schools had a 100% school response rate. All third-year students were eligible and invited to complete the survey, with a 67% student response rate from dental schools; 64% from medical schools; 83% from nursing schools; and 71% from schools of pharmacy. On the day of the survey, students who had left the classroom (21 students) or those who were absent for any other reasons were not followed up. From the 2,344 registered eligible students, 1,590 students responded, giving an overall response rate of 68%.

The survey was an anonymous, modified self-reported questionnaire originally developed by the Tobacco Free Initiative and the World Health Organization (WHO) in collaboration with the US Centers for Disease Control and Prevention (CDC). The questionnaire used was originally in English, and later translated into Arabic by an expert team from the Sudan Ministry of Health (SMOH).

The Sudanese research coordinators, including the author of this study, trained the data collectors from the Ministry of Health, and supervised the data collection. The survey was first piloted among 50 non-Health Science students to determine clarity of questions and to ensure feasibility of administration and the accuracy of translation. After incorporating the corrections, the process resulted in removing, adding or changing some questions, resulting in a final set of 44 questions. These were then back-translated into English, by an independent professional person who was not part of the first translation or study, to check for validity and to avoid bias and/or misunderstanding. The questionnaires were distributed during regular lectures and class sessions. Completion time was about 40–45 min.

### Ethical Approval

Permission to conduct the study was sought from the Ministry of High Education and the Ministry of Health Research Ethics Board, the university authorities' board, and the Dean of each school. The entry point for conducting the student survey for each school was through a designated faculty member to identify the student subject pool. Informed consent was obtained for all eligible students and for each student. This process involved explaining the purpose of the research, and why they had been selected. Their consents were obtained verbally; no written permission was required of individuals at the age of 18 and above, for those who were younger than age 18, parental consents were obtained. Participation was voluntary and confidential, with the option of declining without penalty or loss of benefits to which the student would be otherwise entitled.

### Definitions of Variables and Outcome Measures

The selected questions for this study included demographic characteristics, questions related to school policy and use of three types of tobacco products (cigarettes, shisha and tombak), and a final question related to smoking in places where students live.

#### Tobacco Products

***Cigarettes*** are the most common type of tobacco used throughout the world. Although cigarettes come in a variety of strengths and styles, the questions on the survey did not differentiate type of cigarettes smoked.

***Tombak*** is a loose, moist form of smokeless tobacco. The plant is of the species *Nicotiana Rustica* and/or *glauca*, with a high content of nicotine. *Soute* /saΩt/ is another local name for tombak. The leaves are ground for maturation for up to 1 year for uniform drying and storage for aging (30). Then they are mixed with “*Atrun or Natrun*,” which is a kind of raw alkaline material, consisting of naturally occurring chemical substances, including sodium bicarbonate. The ingredients are then mixed with water and rubbed by hand for blending and constantly tested with the tips of the fingers in a process called “*Tatmeer* /*ta:tæm.i*^*r*^/.” The final product “tombak” will be ready for dipping after many hours (up to 1-day) of being sealed in an airtight container. tæm tiǝr tiǝr tiǝr Tombak is highly addictive because of its high nicotine content. Its use results in serious health problems that are related to several forms of oral cancer (10). The dip in Sudan is named *suffa* /sǝ’f’fǝ/, which is a small moist lump/ball that is made by rolling a small amount of tombak repeatedly on the palm using the thumb, index, and middle fingers of the other hand in a circular manner. *Suffa* is usually placed in the lower labial groove between the lower lip and the gum (*usual site of cancer in Sudan*), or under the tongue or upper lip.

***Shisha*** is another name for “oriental water-pipe” that is well known and popular in the Middle East and North Africa. Shisha is an instrument used for smoking, consisting of a head, body, water bowl, and rubber-pipe (hose). The smoking involves heating the fruit-flavored tobacco, usually with charcoal, cooled down by passing through the water chamber at the bottom of shisha prior to inhalation. Users believe that shisha is safer than cigarettes because the smoke passes through water before inhalation (9). Shisha use is becoming increasingly popular in Sudan among young people sitting for hours at Internet cafes. Its use is also increasing among young girls and women in some beauty centers and hair styling saloons (unpublished document, SMOH, 2007).

#### Tobacco Use Variables

***Ever tobacco user*** included anyone who tried any kind of tobacco products, those who had smoked, dipped tombak, or used shisha at least one time, during the course of her/his life. Each question in these categories was treated independently. The question was “*Have you* ***ever*** *tried or experimented with cigarette smoking/shisha/tombak dipping, even if it was one puff/dip or two?*” Responses were “Never”/“Yes.”

***Current smoking*** question was “*During the past 30 days, on how many days did you smoke cigarettes*?” Students were classified as “current smokers” if they reported smoking on any day of the previous month. Questions about current use of other kinds of tobacco (*shisha and tombak*) were not available in the survey therefore were not measured.

#### School Smoking Policy Variables

For ease of use, the term “school” was used in the text and the tables to refer to all colleges; faculties; and school under study (Dental schools, Colleges of Medicine, Nursing schools, and Faculties of Pharmacy).

##### Smoking on school premises

***Smoking on schools' premises*** question was “*Have you smoked cigarettes on school premises during the last 1-year?”* Responses were “Not-smoked”/“smoked.”

***Dipping on schools' premises*** question was “*Have you dipped tombak on school premises during the last 1-year?”* Responses were “Not-dipped”/“dipped.”

Two additional variables were created from the above two questions, included only the users (the current smokers and tombak dippers) who responded to the questions; and only those who reported “Yes” they had smoked or dipped on school premises were counted.

***Smokers who smoked on school premises*** included both current and ever smokers who smoked during the prior 1-year.

***Current smoking on school premises*** includes only current smokers who smoked on school premises during the prior 1-year.

***Ever dippers on school premises*** includes only those who dipped tombak for the first time on school premises during prior 1-year. The variables about school policy were based on the responses to two questions; the answers were further categorized for purpose of the analysis.

##### Policy banning smoking

(PBS) “Does your school have an official policy banning smoking in school buildings and clinics?” Responses were “No official policy”/“exists in either of them”/“exists in both.”

##### Smoking restriction

*Which of the following best describes your school's official smoking policy for public places or common areas “lobbies, restrooms & dining areas”?* Responses were “No official policy”/“complete restriction”/“partial restriction.”

***Complete restriction:*** Smoking not allowed in any of the mentioned places.

***Partial restriction:*** Smoking is allowed in one of the mentioned places.

#### Residential Smoking Variable

The residential smoking question was “*Has anyone smoked in your presence in places where you live, on one or more days during the last 7-days*?” “No” = 0 days, “Yes” = 1–7 days. The terms of “residential smoking” and “smoking where student lives” are used interchangeably throughout this paper.

### Data Analyses

Pearson's χ^2^ test was performed to examine the descriptive analysis for frequencies, differences in proportion, and associations between variables. Statistical significance is reported two sided: *p* < 0.05. IBM SPSS Statistics version 23.0 was used for the preliminary analysis.

Due to the hierarchical and the tiered nature of the data, multilevel logistic regression was used to examine the relationship between school policy and residential smoking (*exposure*) and the use of tobacco by students (*outcome*). The dataset was assumed to have a two-level data structure, where individual cases (students) are nested in a higher-level group (schools). The individual-level of students' behavior related to tobacco use (*smoking cigarettes, dipping tombak, shisha, and “smoking and dipping” on school premises*) was nested at a higher-level group (25 schools) that simultaneously included policy and residential smoking in the multivariate model with the students as the unit of analysis.

Before conducting the analysis, the outcome tobacco variables were dichotomized. The data were exported to Statistical Package for Stata' gllamm (Generalized Linear Latent and Mixed Stata/SE 14.0) for multilevel logistic regression to fit random-intercept logistic models and to determine the strength of the hypothesized relationships between school policy and student tobacco behavior. A *null model* was first fitted and included only a random intercept to estimate the variations in the use of tobacco across schools (this model is not presented in the tables). A *subsequent unadjusted model* was separately fitted for each smoking policy in relation to tobacco use outcomes. A *final adjusted model* was fitted, adjusted for age and sex, with school level as a random effect. Student reporting about smoking on school premises was analyzed separately. The odds ratios for the fixed part of the models were estimated using 95% confidence intervals. To estimate the differences between schools, the school level variance (SE) were also measured.

## Results

### Descriptive Analysis

Tables 1, 2 provide a summary of the numbers and percentages arranged by tobacco use. Students' reporting about schools' smoking policies are presented as policy banning smoking and restriction, together with exposure to residential smoking. Sample sizes differ slightly among the analyses due to differences in the patterns of missing data.

**Table 1:** Percentage of tobacco users by age, sex, type of school, school smoking policies, and residential smoking.

		**Ever smoking**	**Ever use Shisha**	**Ever dipping tombak**	**Current smoking**	**Smoking on school premises during last 1-year**
	**Grand total** ***N* (%)^▾^**	***N©=* 1570** ***n*^¶^ (%)**	***N©=* 1587** ***n* (%)**	***N©=* 1583** ***n* (%)**	***N*©= 1574** ***n* (%)**	***N*©= 1545** ***n* (%)**
**ALL**	(1590)	641(40.3)	536 (33.7)	256 (16.2)	130 (8.2)	163 (10.5)
**SEX**	***N*****: 1547**					
*Female*	1051 (66)	307 (30)	239 (23)	91 (9)	18 (2)	68 (7)
*Male*	496 (31)	320 (65)	286 (58)	158 (32)	110 (23)	94 (19)
***P-value***		**<0.001**	**<0.001**	**<0.001**	**<0.001**	**<0.001**
**AGE**	***N*****: 1506**					
* <19 years' old*	77 (5)	23 (30)	18 (24)	7 (9)	1 (1)	2 (3)
*19 – 24*	749 (47)	260 (35)	206 (28)	103 (14)	44 (6)	59 (8)
*>24*	680 (43)	317 (47)	278 (41)	129 (19)	76 (11)	83 (12)
***P-value***		**<0.001**	**<0.001**	**0.006**	**<0.001**	**<0.05**
**SCHOOL**	***N:*** **1590**					
*Dental*	193 (12)	92 (48)	87 (45)	29 (15)	19 (10)	27 (14)
*Medical*	711 (45)	313 (45)	237 (33)	113 (16)	58 (8)	77 (11)
*Nursing*	319 (20)	96 (30)	90 (28)	51 (16)	16 (5)	27 (9)
*Pharmacy*	367 (23)	140 (39)	122 (33)	63 (17)	37 (10)	33 (9)
***P-value***		**<0.001**	**0.002**	**0.905**	**0.078**	**0.056**
**SCHOOLS' POLICIES**						
**Official policy banning smoking**	***N*****: 1572**					
*No official policy*	863 (54)	307 (36)	232 (27)	115 (13)	51 (6)	64 (8)
*Exists in either school or clinic*	349 (22)	157 (46)	138 (40)	72 (21)	32 (9)	49 (14)
*Exists in both*	360 (23)	169 (48)	160 (44)	66 (18)	44 (12)	49 (14)
***P-value***		**<0.001**	**<0.001**	**0.008**	**<0.001**	**<0.001**
**Smoking restriction**	***N*****: 1561**					
*No Official Policy*	865 (54)	310 (36)	240 (28)	122 (14)	43 (5)	70 (8)
*Complete restriction*	469 (30)	210 (46)	177 (38)	88 (19)	47 (10)	60 (13)
*Partial restriction*	227 (14)	108 (48)	109 (48)	42 (19)	39 (17)	30 (13)
***P-value***		**<0.001**	**<0.001**	**0.054**	**<0.001**	**<0.05**
**RESIDENTIAL SMOKING**	***N*****: 1573**					
*Not exposed*	944 (59)	310 (33)	253 (27)	123 (13)	35 (4)	70 (8)
*Exposed*	629 (40)	320 (51)	279 (44)	129 (21)	93 (15)	91 (15)
*P-value*		<0.001	<0.001	<0.001	<0.001	<0.001

***▾*N:***Grand Total. Total for each variable varies due to missing data*.

***N***©: *Total number of students responded to the question from the total participants (1590)*.

**¶** ***n***:*number of tobacco users “Yes%” answers*.

**Table 2:** Percentages of students' awareness of school smoking policies as well as residential smoking by basic demographic characteristics and school type.

	**SCHOOL SMOKING** **POLICY^∑^**						**EXPOSURE TO RESIDENTIAL** **SMOKING^¥^**
	**Policies banning smoking in clinics and school buildings** ***N*** **= 1572**			**Smoking restriction in public places and common areas** ***N*** **= 1561**			***N* = 1573**
	**Policy not exists** ***n***^¶^ **(%)**	**policy exists either of them** ***n* (%)**	**policy exists in both of them** ***n* (%)**	**Policy not exists** ***n* (%)**	**Complete^a^ restriction** ***n* (%)**	**Partial**^b^ **restriction** ***n* (%)**	**People smoke in places where participant lives** ***n* (%)**
**ALL^β^**	863 (54)	349 (22)	360 (23)	865 (54)	469 (30)	227 (14)	629 (40)
**SEX**	**839 (54)**			**848 (55)**			
*Female*	640 (61)	204 (20)	197 (19)	628 (60)	266 (26)	141 (14)	347 (33)
*Male*	199 (41)	134 (27)	157 (32)	220 (45)	184 (38)	83 (17)	269 (55)
***P*****-value**		≤0.001	≤0.001		≤0.001	≤0.001	≤0.001
**AGE**	**826 (55)**			**830 (56)**			
* <19*	40 (52)	23 (30)	14 (18)	38 (51)	22 (29)	15 (20)	25 (33)
*19–24*	427 (57)	147 (20)	168 (23)	434 (59)	209 (28)	93 (13)	287 (39)
*>24*	359 (53)	154 (23)	158 (24)	358 (53)	206 (31)	108 (16)	283 (42)
***P*****-value**		0.187	0.198		0.112	0.117	0.184
**SCHOOLS^∞^**	**863 (55)**			**865 (55)**			
*Dental*	48 (44)	45 (23)	63 (33)	99 (53)	42 (22)	48 (25)	86 (46)
*Medical*	416 (59)	156 (22)	134 (19)	417 (60)	174 (25)	107 (15)	289 (41)
*Nursing*	172 (54)	77 (24)	68 (22)	170 (54)	112 (36)	33 (11)	114 (36)
*Pharmacy*	191 (53)	71 (20)	95 (27)	179 (50)	141 (39)	39 (11)	140 (39)
***P*****-value**		≤0.05	≤0.001		≤0.001	≤0.001	0.154

∑ *School policies were examined by students' awareness of banning smoking in clinics and/or school buildings and restriction in public places and common areas (lobbies, restrooms and dining areas)*.

a **Complete restriction**: *smoking not allowed in any public or common areas*.

b **Partial restriction**: *smoking allowed in either common rooms or public places*.

¥ *Residential smoking: is exposure to smoking in places where student lives*.

¶ ***n***:*number of tobacco users, “Yes%” answers*.

β *Total for each variable varies due to missing data*.

∞ **School, faculty and college (***Dental school, College of Medicine, Nursing school and Faculty of Pharmacy). For ease, only (school) will be used in the text*.

The 1,590 participants included more females (66%) than male students, the majority were between the ages of 19 to 24 years. Overall prevalence of ever smoking was 40.3%, more among males than females. Ever smoking, increased with age and varied between schools (ranging from 30 to 48%). The pattern was similar for shisha use. Overall prevalence of shisha use was 33.8%, dippers (16.2%) and current smokers (8.3%), more among male students. Strikingly, 10.5% of all students smoked on school premises. They were also more male students, older, and from dental schools. Those who reported that a policy existed and that smoking was restricted were more likely to smoke on school premises (Table 1).

Most of ever smokers (52%), shisha users (44%), dippers (21%), and 15% of current smokers were exposed to residential smoking on any days of the previous 1 week (Table 1). That means 40% of all participants were exposed to smoking where they live; more than half were male students (55%) and older (42%) (Table 2).

As shown in Table 2, more than half the students reported the non-existence of school smoking policy (54%), and there were no big differences in the percentages between schools. As reported, more nursing students than the rest reported that smoking was completely restricted in public places and common areas (39%).

### Multi-Level Analysis

#### School Smoking Policy

As indicated in Table 3, the existence of policy was significantly associated with ever smoking and shisha use after adjusting for age and sex. Odds ratios declined but remained significant compared to non-smokers, with small reduction of school variance. Partial smoking restriction was significantly associated with increased risk of current-smoking (Adjusted OR (A-OR) 2.91), and shisha use (A-OR 2.17), with small reduction of school level variance. School policy and smoking restrictions appeared to be unrelated to dipping behavior.

**Table 3:** Odds ratios (95% confidence intervals) for tobacco use by school smoking policies and exposure to residential smoking in unadjusted and adjusted models; adjusted for age, sex, and school level as random effects.

	**Ever smoking**		**Current smoking**		**Ever shisha**		**Ever dipping tombak**	
	**Unadjusted**	**Adjusted^ϕ^**	**Unadjusted**	**Adjusted^ϕ^**	**Unadjusted**	**Adjusted^ϕ^**	**Unadjusted**	**Adjusted^ϕ^**
	**OR (95% CI)**	**OR (95% CI)**	**OR (95% CI)**	**OR (95% CI)**	**OR (95% CI)**	**OR (95% CI)**	**OR (95% CI)**	**OR (95% CI)**
**SCHOOL SMOKING POLICIES**								
**Policy banning smoking**								
*No official policy*	1	1	1	1	1	1	1	1
*Policy exists for either of them*	1.53 (1.17–2.01)	1.24 (0.92–1.67)	1.52 (0.94–2.48)	0.95 (0.55–1.64)	1.80 (1.37–2.38)	1.52 (1.12–2.07)	1.71 (1.22–2.40)	1.24 (0.86–1.80)
*Policy exists for both*	1.67 (1.27–2.18)	1.34 (0.99–1.80)	2.14 (1.36–3.36)	1.34 (0.81–2.23)	2.18 (1.66–2.87)	1.80 (1.33–2.45)	1.48 (1.05–2.09)	1.04 (0.72–1.52)
*P*–value	≤0.001	0.055	0.001	0.251	<0.001	<0.001	0.026	0.819
**Random part**								
*School level variance (SE)*	0.16 (0.08)	0.11 (0.07)	0.38 (0.20)	0.19 (0.15)	0.14 (0.07)	0.11 (0.07)	0.08 (0.07)	0.04 (0.05)
**Smoking restriction**								
*No Official Policy*	1	1	1	1	1	1	1	1
*Complete restriction*	1.52 (1.19–1.96)	1.24 (0.94–1.63)	2.17 (1.38–3.41)	1.32 (0.82–2.16)	1.66 (1.28–2.15)	1.36 (1.02–1.80)	1.42 (1.04–1.94)	1.04 (0.73–1.47)
*Partial restriction*	1.54 (1.13–2.10)	1.35 (0.96–1.90)	3.53 (2.18–5.73)	2.91 (1.68–5.02)	2.26 (1.65–3.09)	2.17 (1.54–3.06)	1.39 (0.94–2.06)	1.14 (0.74–1.75)
*P*–value	0.001	0.083	≤0.001	≤0.021	≤0.001	0.034	0.1	0.544
**Random part**								
*School level variance (SE)*	0.16 (0.07)	0.12 (0.08)	0.28 (0.17)	0.08 (0.10)	0.15 (0.08)	0.07 (0.06)	0.07 (0.06)	0.04 (0.05)
**EXPOSURE TO RESIDENTIAL SMOKING**								
*Not exposed*	1	1	1	1	1	1	1	1
*Exposed*	2.16 (1.75–2.68)	1.66 (1.31–2.10)	4.45 (2.95–6.69)	3.12 (1.98–4.92)	2.16 (1.74–2.68)	1.73 (1.36–2.21)	1.72 (1.31–2.26)	1.33 (0.98–1.80)
*P*–value	≤0.001	≤0.001	≤0.001	≤0.001	≤0.001	≤0.001	≤0.001	0.069
**Random part**								
School level variance (SE)	0.16 (0.08)	0.05 (0.05)	0.33 (0.19)	0.19 (0.14)	0.16 (0.08)	0.09 (0.06)	0.06 (0.06)	0.01 (0.04)

*Variance of random effects [SE] for ever smoking (0.17 [0.08]), current smoking (0.38 [0.20]), Ever shisha (0.18 [0.09]) and Ever dipping tombak (0.07 [0.06])*.

ϕ *Adjusted for age, sex and school level as a random effect*.

A third model was fit and further adjusted for school types and residential smoking as confounders in addition to age and sex. The adjusted odds ratio remained almost the same when adjusted for school types, but when adjusted for residential smoking, the odds ratio decreased and the results lost their significances. The results of this analysis were not presented in the tables. This may underestimate the importance of school factors influencing students' tobacco use (31).

#### Exposure to Residential Smoking

In the adjusted model, exposure to smoking during the last 7-days was significantly associated with increased risk of ever smoking (A-OR 1.66), current smoking (A-OR 3.12), and shisha use (A-OR 1.73), and marginally (*P*-value 0.06) related to dipping tombak (Table 3).

#### Smoking on School Premises

The results shown in Table 4 are consistent with the previous results of the multi-level analysis. The awareness of an official policy did not appear to stop students from dipping or smoking on school premises, when the students reported the existence of policies, the risk increased for tobacco use (A-OR 2.38, *for current smoking* and 2.60 *for ever dipping)*. Regarding smoking restriction, it was significant only with complete restriction.

**Table 4:** Odds ratios (95% confidence intervals) for tobacco use on school premises during the prior 1–year by school smoking policies and exposure to residential smoking among smokers and tombak dippers; adjusted for age, sex, and school level as random effects.

	**Smoking on school premises during prior 1–year^a^** ***N*** **= 163**		**Current smoking on school premises prior 1–year^b^** ***N*** **= 77**		**Ever dipping on school premises during the prior 1–year^c^** ***N*****= 56**	
	**Unadjusted OR (95% CI)**	**Adjusted^ϕ^ OR (95% CI)**	**Unadjusted OR (95% CI)**	**Adjusted^ϕ^ OR (95% CI)**	**Unadjusted OR (95% CI)**	**Adjusted^ϕ^ OR (95% CI)**
**SCHOOL SMOKING POLICIES**						
**Policy banning smoking (for school building and clinics)**						
*No official policy*	1	1	1	1	1	1
*Policy exists for either*	2.04 (1.36–3.08)	1.92 (1.23–3.01)	0.22 (0.07–0.65)	0.23 (0.08–0.67)	3.51 (1.73–7.14)	2.32 (1.06–5.12)
*Policy exists for both*	1.98 (1.31–2.99)	1.63 (1.04–2.59)	2.58 (1.50–4.44)	2.38 (1.34–4.25)	3.71 (1.86–7.41)	2.60 (1.22–5.56)
*P*–value	0.001	0.034	0.001	0.003	<0.001	0.013
**Random part**						
*School level variance (SE)*	0.13 (0.11)	0.14 (0.13)	1.59 (0.80	1.54 (0.79)	0.22 (0.22)	6.84 (1.39)
**Smoking restriction**						
*No official policy*	1	1	1	1	1	1
*Complete restriction*	1.75 (1.20–2.59)	1.59 (1.04–2.43)	1.31 (0.74–2.30)	1.38 (0.77–2.47)	2.88 (1.56–5.30)	2.30 (1.16–4.53)
*Partial restriction*	1.61 (1.01–2.60)	1.63 (0.99–2.70)	1.33 (0.64–2.75)	1.30 (0.61–2.80)	1.62 (0.69–3.82)	1.60 (0.64–3.97)
*P–*value	0.046	0.055	0.441	0.489	0.001	0.311
**Random part**						
*School level variance (SE)*	0.18 (0.14)	0.13 (0.13)	1.46 (0.74)	1.40 (0.72)	0.22 (0.22)	4.48 (3.99)
**EXPOSURE TO RESIDENTIAL SMOKING**						
*Not exposed*	1	1	1	1	1	1
*Exposed*	2.10 (1.51–2.95)	1.45 (1.00–2.10)	0.94 (0.57–1.53)	0.85 (0.50–1.44)	2.18 (1.26–3.79)	1.35 (0.74–2.49)
*P–value*	<0.001	≤0.05	0.791	0.554	≤0.05	0.324
**Random part**						
*School level variance (SE)*	0.18 (0.14)	0.16 (0.14)	1.58 (0.78)	0.50 (0.38)	0.36 (0.31)	7.80 (1.66)

*Variance of random effects [SE] Ever smoked at school (0.24 [0.16]) smoking in school prior 1–year (0.20 [0.14]), Current smoking in 1–year (1.39 [0.68]) Tombak (0.30 [0.28])*.

a *Included only those who smoked: students who either ever or current smokers (total = 513)*.

b *Those were only current smokers who smoked on school premises = 77 students constitute 59.2 %, of all current smokers (total = 130 see Table 2)*.

c *Those were only dippers who dipped on school premises = 56 students constitute 21.9% of all dippers (total dippers = 256 dippers, see Table 2)*.

ϕ *Adjusted for age and sex and school level as a random effect*.

## Discussion

The present study reveals new information about the pattern of tobacco use among Sudanese university students and its association with school anti-smoking policies. The study makes an important contribution to what is known about the tobacco habits of youth by including tombak dipping and shisha use in addition to cigarette smoking. No known study to date has been published in which the researcher compared these three types of tobacco use among youth or has considered the effect of school smoking policy on dipping tombak and/or using shisha and the effect of residential smoking on using different kinds of tobacco products.

Current smoking was observed in 8.3% of the Health Sciences students, compared to 13.7% among university students in Sudan in a study conducted in 2016 (32). Another study conducted in 2013 among 302 Sudanese Health Professional students from private schools (33) showed the prevalence of current smoking and ever smoking to be 9.6 and 26%, respectively, and 34.5% were exposed to residential smoking (compared to 40.8% of the present sample). Comparing the findings of current smoking from the three studies shows no significant reduction among current smoking in terms of policy measures, and strategic efforts to prohibit smoking among students during the period in between the three studies (2007, 2013, and 2016) do not appear to have been effective.

Regarding the students' reports about school policy, the results demonstrate a considerable diversity between schools. The kinds of tobacco product used were quite different among students. More than half of all students reported the non-existence of school policy (54%). Most of the tobacco users were aware of the existence of policy and the restriction of smoking in common areas and in school buildings. It would seem that tobacco users are more aware of school smoking policies than non-users, even though they may choose to ignore the policies.

With additional analysis, the awareness of existence of school policies diminished and lost its significant results except for shisha users. On the other hand, awareness of smoking restriction was significantly and reversely associated with increasing risk of current smokers and with shisha users. This implies either the policy was not well-enforced (26), or there was a lack of student commitment to school policy (34).

With respect to the shisha use category, shisha users were more aware of the policy existence and the restricted areas. That might be because it is inconvenient to use shisha inside a school compared to cigarette smoking and dipping. The size of the shisha, the strong smell of fruit-flavored tobacco, and the fact that shisha is usually smoked in groups (35) would collectively hinder the use of shisha inside the school. In addition, there is lack of literature about policy concerning shisha use (36). In contrast, the “dip” can be hidden behind the lips and remain hidden for several minutes (14), which makes it easier for dippers to dip inside schools. Unlike smoking cigarettes or using shisha, dippers were more aware of smoking and restriction zones only if they reported being dual users (37).

With regard to smoking on school premises, 31.8% of all smokers and 21.8% of dippers have smoked or dipped on school premises (Table 4, footnote). Specifically, more than half of the current smokers (59.2%) have smoked inside the school they attend (Table 4, footnote). Results of multi-level analysis showed that despite knowing the existence of a non-smoking policy, students continued to smoke and dip on school premises, meaning that the awareness of the policy had no effect on their smoking or dipping, or they knew that it was not vigorously enforced.

Students' reports about their school policy is quite ambiguous. Their responses varied greatly, despite the fact that they may be in the same schools (31, 38). Awareness of the policy and enforcement are essential to help ensure compliance (39). Various researchers have shown that the relationship between school policies and students' -smoking are mixed (20, 40). Some indicate a weak-to moderate relationship between policies and student smoking, while other studies indicate no effects (41). Other researchers suggest that changing a school environment represents a broader yet appropriate and effective factor in prevention and protection (26, 42).

More than half of ever smokers and 44% of shisha users; at least on 1 day of the previous week, were exposed to smoking in places where they are living, more among older male students (55%). Those exposed to residential smoking were more susceptible to start smoking, and they were more than three times at risk to be current smokers and twice as likely to be shisha users (14). Exposure to residential smoking did not affect a student's risk of dipping tombak, and it was also not found to be related to smoking on school premises. Most researchers who examined smoking among young revealed that ever smokers initiated their smoking early in adolescence (43, 44), raising a question of the possibility of exposure to residential smoking perhaps some time before getting into their current university. Therefore, it made it crucial, in the present study, to consider the exposure to residential smoking as one of the main factors behind starting to use cigarettes when students were already adults. Accordingly, this finding itself is an indicator of many other unmeasured influences on student tobacco use. Further research in the low- and middle-income countries is needed to explore other factors, possibly imitating friends (45) or socio-economic background (46), or exposure to smoking in places other than the places where they live.

Tombak dipping is a normative behavior among Sudanese. The product is cheap, easily available, and widely used. Some smokers have adopted it as an alternative to smoking tobacco (14). Results of the present study indicated that tombak is less used among Health Science students (16%) compared to the 45% prevalence of tombak among the adult population (47). This might be explained in that tombak is less prestigious among young university students and is less accepted, especially among females.

### Strengths and Limitations

This study makes an important contribution to researches about school tobacco policies in Africa. The findings could be taken as a baseline for school policies in Sudan and other Sub-Saharan countries targeting the same group, although it was carried out in a country which is now two separate countries. Several factors may cause different students in different schools in different countries to experience the effect of tobacco use in different ways, but a baseline is important for the future researchers to measure the effect of changes over time. One of the most important strengths of the present study is that the researcher considered different kinds of tobacco products, and similarly assessed use of those products. By understanding the use of varied tobacco products and their associations with school policy, the results can be used to target future policy programs that address use of different kinds of tobacco products in the developing world and in establishing a school-based tobacco policy. Another important strength of the present study was the use of a standardized questionnaire for data collection; it was based on the international core questionnaire. The original questionnaire was translated by expert persons, piloted, and back-translated by an independent professional person who was not part of the first translation or the study to minimize bias and misunderstanding. More questions were also asked about local tobacco products. The overall response rate of school was 100%, and 68% for students, yet non-response bias might have affected the results. Some researchers have found that non-respondents are more likely to be smokers than respondents (48), the author does not expect that non-response rate (32%) of the students had a major role on influencing the results of this study.

The most important limitations of this study included its cross-sectional design (49); therefore, a causal relationship cannot be determined. For instance, from the findings obtained in this study, the direction of causality of the association between tobacco use by students and their attitudes toward anti-smoking policy was not clear. Longitudinal studies are required to make the direction of this kind of causality clear. The data for this study were only collected from third year Health Sciences Students; therefore, it cannot be assumed that the sample is representative of other students at the same age at other universities or in other academic disciplines of study. Other measures, such as school written policies from the schools, to validate the students' reports were not available. More information would be valuable in supporting the findings, since the official written policy and the students' perception of policy might be different. The data used in the present study were collected in 2007. To conduct another study; targeting the same study population would not be easy because the country has subsequently divided into two countries. However, findings from data collected among some Health Science students in the northern part of Sudan in 2013 and 2016 (32, 33), showed that situation about tobacco use is worse than in 2007.

## Conclusions

Despite the limitations, the results of this study provide important information as research about use of tobacco products continues. The present study was designed to examine the relationship between tobacco control activities and tobacco use prevalence among young adults, confirming the importance of continuous review of policies and prevention programs. Although school policies banning or restricting tobacco use seemed to be largely ignored, no specific elements with significant effects were found in the school context that would lead to specific suggestions for improvement of the policies. Yet, strategies for tobacco control must be extended to cover school, societal, and individual levels. From a research perspective, the high prevalence of tobacco use by the study population calls into question what other factors in the environment, including cultural norms, seem to promote smoking and result in such a high percentage of future health professional being tobacco users.

## Recommendations

Based on the findings from this study, opportunities for schools to have a significant role in preventing tobacco use should be pursued. Further work is needed with emphasis on students' tobacco use attitudes and behavior for every form of tobacco product. More attention should be given to enforcement of school smoking policies, to help dissuade smoking initiation. Future researchers who use the questionnaire from this study in school settings should include a random sample of students from preparatory to finalist classes, representing a wider life span and geographical representation than in the present study. Researchers studying tobacco use and prevention should examine all major kinds of tobacco products, as the results here indicated that use of different types of tobacco is associated with different individual characteristics and environmental contexts. Different stages in a student's life should also be considered, including the socio-cultural background of the student, the school environment, residential and neighborhoods' smoking, and financial accessibility that might have effect on a student's initiating or continuing use of any kind of tobacco product.

## Data Availability Statement

The datasets generated for this study are available on request to the corresponding author.

## Author Contributions

SE conceived the study, collected the data, conducted the analysis, drafted, and finalized the manuscript.

### Conflict of Interest

The author declares that the research was conducted in the absence of any commercial or financial relationships that could be construed as a potential conflict of interest.
